# Resilience and MRI correlates of cognitive impairment in community-dwelling elders

**DOI:** 10.1192/bjp.bp.114.152363

**Published:** 2015-11

**Authors:** Anya Topiwala, Charlotte L. Allan, Vyara Valkanova, Enikő Zsoldos, Nicola Filippini, Claire E. Sexton, Abda Mahmood, Archana Singh-Manoux, Clare E. Mackay, Mika Kivimäki, Klaus P. Ebmeier

**Affiliations:** **Anya Topiwala**, BMBCh, **Charlotte L. Allan**, MBChB, **Vyara Valkanova**, MD, **Enikő Zsoldos**, MSc, Department of Psychiatry, University of Oxford, Warneford Hospital, Oxford, UK; **Nicola Filippini**, DPhil, **Claire E. Sexton**, DPhil, Department of Psychiatry, University of Oxford, Warneford Hospital, Oxford, UK and Oxford Centre for Functional MRI of the Brain (FMRIB), Nuffield Department of Clinical Neurosciences, University of Oxford, Oxford, UK; **Abda Mahmood**, MSc, Department of Psychiatry, University of Oxford, Warneford Hospital, Oxford, UK; **Archana Singh-Manoux**, PhD, Department of Epidemiology and Public Health, UCL, London, UK and INSERM, U1018, Centre for Research in Epidemiology and Population Health, France; **Clare E. Mackay**, PhD, Department of Psychiatry, University of Oxford, Warneford Hospital, Oxford, UK; **Mika Kivimäki**, PhD, Department of Epidemiology and Public Health, UCL, London, UK; **Klaus P. Ebmeier**, MD, Department of Psychiatry, University of Oxford, Warneford Hospital, Oxford, UK

## Abstract

**Background**

The contribution of education and intelligence to resilience against age-related cognitive decline is not clear, particularly in the presence of ‘normal for age’ minor brain abnormalities.

**Method**

Participants (*n* = 208, mean age 69.2 years, s.d. = 5.4) in the Whitehall II imaging substudy attended for neuropsychological testing and multisequence 3T brain magnetic resonance imaging. Images were independently rated by three trained clinicians for global and hippocampal atrophy, periventricular and deep white matter changes.

**Results**

Although none of the participants qualified for a clinical diagnosis of dementia, a screen for cognitive impairment (Montreal Cognitive Assessment (MoCA) <26) was abnormal in 22%. Hippocampal atrophy, in contrast to other brain measures, was associated with a reduced MoCA score even after controlling for age, gender, socioeconomic status, years of education and premorbid IQ. Premorbid IQ and socioeconomic status were associated with resilience in the presence of hippocampal atrophy.

**Conclusions**

Independent contributions from *a priori* risk (age, hippocampal atrophy) and resilience (premorbid function, socioeconomic status) combine to predict measured cognitive impairment.

One in nine people worldwide is 60 years or older, and this proportion is projected to increase to one in five by 2050.^1^ With a prevalence of dementia between 6 and 7% of over 65-year-olds,^2^ cognitive decline in ageing populations is creating an increasing social and financial challenge, although traditional estimates may overestimate the detrimental effects of age in the contemporary population.^2–4^ Advanced imaging techniques, such as magnetic resonance imaging (MRI), allow for detailed investigation of the ageing brain. Visual inspection of structural MRIs is a quick and reliable technique that does not require specialist software pipelines or expertise, and is routinely used in clinical practice, unlike automated analysis. Visual inspections can be used to detect atrophy in the grey matter (particularly the medial temporal lobe), and hyperintensities in white matter, which have been highlighted as correlates of functional impairment and dementia.^5,6^ In people over 60 years, such minor MRI abnormalities are, however, often characterised as ‘normal for age’. However, the functional implications of these changes identified by visual inspection are unclear; hence it is difficult for the clinician to ascribe significance to them. Moreover, apart from a few exceptions,^7^ studies of cognitive deficits have ignored factors that may confer functional resilience against structural brain damage.

This paper describes the cognitive profile and routine MRI findings from the first quarter of the Whitehall II imaging substudy of 800 participants.^8^ This sample was recruited from the Whitehall II occupational cohort of 6035 civil servants from 20 UK Government departments in London.^9^ Among 208 participants aged 60–82, we examine the relationship between MRI abnormalities, often described as age-related, and performance on tests estimating premorbid intelligence and cognitive impairment, in relation to factors that may confer resilience against cognitive impairment, including education and premorbid IQ. We define resilience as the positive effect of variables on cognitive outcome given a certain severity of risk factors or organic changes of the brain. Our hypotheses were that cognitive impairment measured by the Montreal Cognitive Assessment (MoCA) would be associated with brain abnormalities, in particular hippocampal atrophy and deep white matter changes, after controlling for confounders, such as age, gender, education and premorbid IQ. At the same time, we predicted that with a given degree of brain abnormality (hippocampal atrophy or deep white matter changes) and other confounders being equal, higher premorbid IQ and education would predict a higher MoCA score, i.e. a smaller chance of cognitive impairment.

## Method

### Participants

The Whitehall II study was established in 1985 at University College London, and recruited 10 308 non-industrial civil servants across a range of employment grades. Eight hundred of these were randomly selected for the current Whitehall II imaging sub-study,^8^ from a cohort of approximately 6035 community-dwelling elders (29 were oversampled from participants previously scoring higher (score ≥16) on the Centre for Epidemiologic Studies Depression (CES-D) scale and are included in this study). This paper describes results from the first 208 participants recruited to the imaging substudy. Participants gave informed consent and attended the investigation in Oxford, unless MRI was contraindicated.

### Magnetic resonance imaging

MRI scans were acquired at the University of Oxford Functional Magnetic Resonance Imaging of the Brain (FMRIB) Centre, using a 3 Tesla Siemens scanner (see online supplementary materials and protocol paper^8^ for further details). Images from the T1-weighted and FLAIR (fluid-attenuated inversion recovery) sequences were used for visual inspection.

### MRI analysis

Scans were assessed independently by three medically qualified researchers (A.T., C.L.A. and V.V.) trained in visual inspection techniques, masked to behavioural details and participant identity for: global atrophy, hippocampal atrophy and white matter changes. Global atrophy was assessed viewing supra-ventricular axial slices and rated from absent (0) to severe (3). Standards for each grade had been agreed in advance in consultation with a fourth researcher with expertise in this field (K.P.E.). Hippocampal atrophy was assessed by the Scheltens scale separately for each side according to the width of the choroid fissure, width of the temporal horn and height of the hippocampus (0–4).^5^ White matter changes were graded by the Fazekas scale depending on the presence and size of deep white matter changes (0–3), and the presence or extent of periventricular white matter changes (0–3).^10^ This scale provides two different scores each, rated on a 4-point scale.

After recording scores separately, disagreements were settled in consultation with a fourth researcher (K.P.E.) and a consensus score reached. Raters remained masked to all other participant data. Intra- (on a random 10% of 208 scans) and interrater reliability (*n* = 208) were assessed by intraclass correlation coefficients (ICCs). For the purpose of the statistical analysis, global atrophy and Fazekas scores were rated as abnormal if >1; hippocampal atrophy was only recorded, if both Scheltens scores were >1.

### Cognitive function

Cognitive function was assessed immediately prior to the MRI scan according to a protocol including paper and pencil instruments based on a systematic review^11^ and extensively piloted in patient groups and healthy volunteers: MoCA, Trail Making Test (TMT A and B), Lexical (letter: ‘F’) and Semantic Fluency (category: ‘Animals’), Rey–Osterrieth Complex Figure (RCF) copying, RCF immediate recall, Hopkins Verbal Learning Test (HVLT-R) immediate recall, Boston Naming Test (BNT), Digit Span and Digit Coding (from the Wechsler Adult Intelligent Scale-IV), Test of Premorbid Function (TOPF), HVLT-R delayed recall and RCF delayed recall (see online supplement for detailed explanation and references). The test battery was administered by trained psychology graduates and psychiatrists.

### Statistical analysis

MoCA scores were modelled by logistic regression, as implemented in SPSS 22 for Windows (IBM Corporation, Armonk, New York, USA). After dichotomising variables at the mean (except for 0–3 MRI scales, where the binary cut-off was between 1 and 2, and for the MoCA, where we used the conventional screening cut-off of 25/26), we entered general atrophy, hippocampal atrophy (only if bilateral), deep white matter changes and periventricular white matter changes separately as independent variables. The resulting odds ratios were compared with odds ratios corrected for age, gender, socioeconomic status, education (years of full-time+half years of part-time education, as required for correction of TOPF) and premorbid IQ estimated from TOPF score alone.

## Results

The mean age of the 208 participants was 69.2 years (s.d. = 5.4), and they were predominantly men 169/208 (81.3%). The imaged sample was representative of the Phase 11 Whitehall cohort for age, body mass index (BMI) and heart rate, had marginally shorter education (95% confidence intervals (CIs) for difference between means: −0.98 to −0.02 years) and lower CES-D scores (95% CI−2.35 to −0.25; see Table DS1 in the online supplement to this paper). Their mean blood pressure was slightly higher (systolic: 95% CI 12.9 to 17.5 mmHg; diastolic: 95% CI 5.8 to 8.6 mmHg). They used more alcohol (95% CI 4.8 to 9.2 units per week). The ratio of men to women was higher in the imaging sample (χ^2^ = 13.78; *P* = 0.0002), and there was an excess of executive and a relatively smaller proportion of clerical civil servants (χ^2^ = 14.51; *P* = 0.0007; d.f. = 2).

**Fig. 1:**
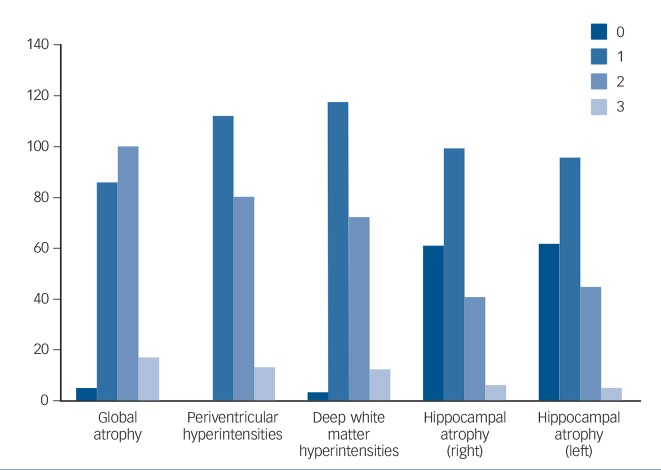
Distribution (histograms) of global atrophy, Scheltens and Fazekas scores.

In general, participants had relatively good cognitive function. Using the conventional cut-offs, 11/208 (5.3%) had an abnormal (<19) score on the HVLT-R; 46/208 (22.1%) scored <26 on the MoCA. The respective normal distribution values, often used as cut-off for normality (i.e. 1 and 1.5 s.d. below the mean) were 24.6 and 23.4 for the MoCA, and 21.7 and 19.2 for the HVLT-R (for details of cognitive tests and the psychiatric diagnoses recorded after Structured Clinical Interview for DSM-IV-TR Axis I Disorders (SCID-1) interview,^12^ see online supplement). Inter- and intrarater reliability for MRI scores was high (ICC 0.8–0.9 and 0.7–0.9 respectively). Scores were approximately normally distributed (Fig. 1), i.e. the majority of participants had higher than minimum (perfect) atrophy and white matter scores.

Participants with high (≥26) and low (<26) MoCA scores were compared for sociodemographic, clinical and cognitive variables (Table 1). Individuals with low MoCA were slightly older (*F*(1,206) = 10.6, *P* = 0.001), there was an over-representation of low MoCA in professional (2nd) and clerical (3rd), as opposed to executive (1st) socioeconomic strata (χ^2^ = 4.5, *P* = 0.03, d.f. = 2), but there were no differences in gender (χ^2^ = 0.07, *P* = 0.79, d.f. = 1), reported minor neurological history (Guillain-Barre Syndrome; brain cyst; transient ischaemic attack; migraine; epilepsy; multiple sclerosis; Parkinsonism; myalgic encephalopathy; blackout; familial tremor; sleep disorder; χ^2^ = 1.63, *P* = 0.20, d.f. = 1), history of major depressive episode (from SCID-1; χ^2^ = 0.002, *P* = 0.97, d.f. = 1) or caseness on CES-D (CES-D≥15; χ^2^ = 1.04, *P* = 0.31, d.f. = 1). There were also no differences in socioeconomic and clinical variables, including alcohol use (Table 1), nor was there a difference in premorbid IQ (*F*(1,206) = 3.3, *P* = 0.07).

**Table 1:** Descriptive variables for high (≥26) and low (<26) MoCA groups

	Low MoCA group (<26)			High MoCA group (526)		
Variable	Mean	s.d.	*n*	Mean	s.d.	*n*
Age, years	**71.3**	**6.1**	**46**	**68.5**	**5.0**	**162**
Alcohol units/week	15.9	15.4	45	16.7	15.8	155
Body-mass index, kg/m^2^	26.3	4.2	46	26.5	4.4	162
Systolic blood pressure, mmHg	145.7	18.3	46	141.8	17.5	161
Diastolic blood pressure, mmHg	77.3	8.9	45	78.5	10.3	161
Heart rate, beats per minute	66.6	11.7	43	67.9	13.3	161
CES-D score	7.5	7.6	46	5.57	6.8	162
Years of education	16.5	4.3	46	15.5	3.3	162
Premorbid IQ^a^	115.6	12.6	46	118.6	8.9	162
MoCA (correct out of 30)	**23**	**2.0**	**46**	**28**	**1.3**	**162**
Boston naming test (correct out of 60)	**54.5**	**8.6**	**46**	**57.8**	**3.2**	**162**
Digit coding (correct out of 135)	**49.3**	**13.3**	**46**	**64.9**	**12.7**	**162**
Digits backward (correct out of 16)	**8.63**	**2.59**	**46**	**10.25**	**2.57**	**162**
Digits forward (correct out of 16)	**10.04**	**2.17**	**46**	**11.16**	**2.26**	**162**
Digits sequence (correct out of 16)	**8.50**	**2.92**	**46**	**10.70**	**2.49**	**162**
Lexical fluency, words per minute	**12.63**	**5.11**	**46**	**16.17**	**4.31**	**162**
Semantic fluency, words per minute	**17.91**	**5.70**	**46**	**22.83**	**5.63**	**162**
Trail Making Test A, seconds	**40.04**	**17.79**	**46**	**29.77**	**10.97**	**160**
Trail Making Test B, seconds	**98.98**	**49.99**	**45**	**58.79**	**22.85**	**160**
HVLT (delayed recall, correct out of 12)	**7.09**	**3.55**	**46**	**9.33**	**2.71**	**162**
HVLT (immediate recall, correct out of 36)	**23.74**	**5.79**	**46**	**27.65**	**4.48**	**162**
RCFT (copy, correct out of 36)	**27.20**	**6.44**	**46**	**30.83**	**3.78**	**161**
RCFT (delayed recall, correct out of 36)	**10.04**	**5.60**	**46**	**15.43**	**5.99**	**161**
RCFT (immediate recall, correct out of 36)	**11.03**	**6.62**	**46**	**15.88**	**6.07**	**161**

MoCA, Montreal Cognitive Assessment; CES-D, Centre for Epidemiologic Studies – Depression; HVLT, Hopkins Verbal Learning Test; RCFT, Rey–Osterrieth Complex Figure Test. Results with *P*< 0.05 are in bold.

a. Test of premorbid function (IQ corrected for gender and education).

Hippocampal atrophy and deep white matter changes (as defined above) were associated with abnormal MoCA scores. Although the mean odds ratio for both general atrophy and periventricular white matter changes were above 1, confidence intervals indicated no significant effect (Table 2). After correction for potential confounders (age, gender, socioeconomic status, years of education and premorbid IQ), only hippocampal atrophy remained associated with abnormal MoCA. In the presence of hippocampal atrophy, higher premorbid IQ and social class (executive rather than professional or clerical) were independently associated with resilience to cognitive impairment.

**Table 2:** Odds ratios for MoCA (≥26/<26) with normal/abnormal MRI measures

Measure	Odds ratios	95% CI	*P*
Uncorrected odds ratio			
≥1 normal hippocampi/both hippocampi abnormal	**3.43**	**1.61–7.31**	**0.001**
No general atrophy/general atrophy	1.83	0.92–3.64	0.09
Normal Fazekas/deep white matter changes	**2.28**	**1.16–4.48**	**0.02**
Normal Fazekas/periventricular white matter changes	1.80	0.92–3.53	0.09
Corrected odds ratios^a^			
≥1 normal hippocampi/both hippocampi abnormal	**2.75**	**1.16–6.50**	**0.02**
Age (higher/lower)	0.63	0.29–1.37	0.24
Premorbid IQ^b^ (higher/lower)	**2.19**	**1.02–4.71**	**0.045**
Gender (female/male)	1.67	0.60–4.64	0.24
Social class (lower/higher)	**0.46**	**0.22–0.99**	**0.048**
Years of education (higher/lower)	0.50	0.22–1.13	0.095

Results with *P*< 0.05 are in bold.

a. Logistic regression with potential predictor and confounder variables: =1 normal hippocampi, age, gender, social class, years of education and premorbid IQ based on Test of Premorbid Function; *n* = 205.

b. Premorbid IQ calculated from Test of Premorbid Function scores without correction for gender and years of education.

## Discussion

We observed a significant number of minor MRI abnormalities, in particular whole brain and hippocampal atrophy, as well as white matter changes (Fig. 1). Direct comparison with other published studies is difficult, given the differing imaging protocols, rating scales and rater expertise. Nonetheless, the Rotterdam scan study, for example, reported a slightly lower prevalence of white matter lesions compared with our findings (92% *v.* 98.5% deep white matter changes, 80% *v.* 100% periventricular white matter changes).^6^ Similarly, hippocampal atrophy in older populations has been reported at lower rates than the 70% we found (e.g. 33%).^13^ This could reflect a true increased burden of pathological changes or increased detection by our higher resolution MRI protocol (all the above studies used a field strength of 1.5T in contrast to 3T in this project).

Compared with previous studies, the proportion of participants with global cognitive impairment was high (20%).^14^ Potential health concerns may have induced some participants to attend the testing, so the potential for selection bias cannot be dismissed, as those concerned about memory problems may have been more likely to attend. No participant had an established diagnosis of dementia, which is unsurprising given the study inclusion criteria (community resident and ability to travel to Oxford). Unlike the original MoCA validation study,^15^ our sample was not a healthy control group but a community sample, which included those with a history of major (17% of sample) and minor (9% of sample) depression or bipolar disorder (1% of sample, see online supplement). Deficits in executive function and attention are known to persist in euthymic patients with a history of unipolar depression^16^ or bipolar disorder,^17^ although there was neither an excess of major depressive disorders nor of current CES-D caseness in the low MoCA group (Table 1). The level of alcohol use in our cohort (mean 16.5 units/week) may also be relevant. Frequent or heavy (>15 units per week) drinkers may be at increased risk of cognitive impairment^18^ and dementia,^19^ as well as increased ventricle and sulcal size,^20^ although there was no difference in alcohol use between high and low MoCA scorers (Table 1).

Our sample was representative of the larger Whitehall II cohort for age, BMI and heart rate, but had a marginally shorter length of full-time education. Although they scored a couple of points lower on the CES-D depression scale, they used 5–10 units of alcohol more than the Phase 11 cohort and had a higher blood pressure. There was an excess of men and of executive civil servants relative to clerical staff. One implication of these differences may be that the imaging cohort was more likely to generate associations relying on variability for cardiovascular risk factors.

Of the clinical MRI measures, only deep white matter changes and hippocampal atrophy were significantly associated with cognitive impairment. After correcting for possible confounder variables, only hippocampal atrophy remained associated with MoCA (Table 2). This supports the notion that MoCA may predict pathological deterioration in memory, rather than representing the normal process in ageing.^21–25^ In contrast, global atrophy and periventricular white matter changes appear to have little impact on cognition, which lends credence to their being reported as ‘normal for age’.^26^ Although a quantitative review found that white matter changes are associated with poorer global cognitive function, speed of processing, immediate-recent memory, delayed memory and executive function,^27^ not all studies have corroborated these findings.^28^ Our finding that deep white matter changes are associated with MoCA, but that this association is lost after correcting for potential confounders, may be due to limited power of a study of even 200 participants.

With a given degree of hippocampal atrophy, higher premorbid IQ and socioeconomic status (based on civil service grade) but not education were independently associated with resilience to cognitive impairment. This lends strength to the cognitive reserve^29^ or compensation hypotheses.^30^ It may also explain why the Whitehall cohort is resilient to functional deterioration (several of the mean test scores are higher than published results at similar ages^14,31,32^ despite more prevalent structural brain changes). This cohort has a higher education level^33^ and a lower cardiovascular risk profile than those in other studies.^34^ Finally, there are a number of other determinants of cognitive reserve not explored in this study, such as participation in leisure activities,^35^ cohesion of social networks,^36^ occupational complexity^37^ and personality characteristics that may be responsible for additional variability.^38^

We were able to combine 3T MRI imaging with comprehensive cognitive testing in a large study drawn from an occupational cohort. Limitations to our study include its cross-sectional design, and further work needs to include longitudinal and diagnostic follow-up data. Although previous work has demonstrated the clinical value of the MRI scales used,^39^ and our interrater reliability figures were higher than those quoted in several other studies,^40^ it will be valuable to compare our results with automated volumetric measurements to establish whether the key findings (e.g. that hippocampal atrophy is highly functionally relevant and premorbid intelligence and social class confer resilience to functional but not structural deterioration) can be corroborated. In the meantime, our results should contribute to the interpretation of ‘age-related’ MRI abnormalities as they are usually reported in clinical practice.
